# Monitoring prescribing patterns using regression and electronic health records

**DOI:** 10.1186/s12911-017-0575-5

**Published:** 2017-12-19

**Authors:** Daniel Backenroth, Herbert S. Chase, Ying Wei, Carol Friedman

**Affiliations:** 1 Columbia University Mailman School of Public Health, 722 West 168th Street, #633, New York, NY 10032 USA; 2 Department of Biomedical Informatics, Columbia University, 622 West 168th Street, PH-20, New York, NY 10032 USA

**Keywords:** Health care quality control, Electronic health records, Prescribing patterns

## Abstract

**Background:**

It is beneficial for health care institutions to monitor physician prescribing patterns to ensure that high-quality and cost-effective care is being provided to patients. However, detecting treatment patterns within an institution is challenging, given that medications and conditions are often not explicitly linked in the health record. Here we demonstrate the use of statistical methods together with data from the electronic health care record (EHR) to analyze prescribing patterns at an institution.

**Methods:**

As a demonstration of our method, which is based on regression, we collect EHR data from outpatient notes and use a case/control study design to determine the medications that are associated with hypertension. We also use regression to determine which conditions are associated with a preferential use of one or more classes of hypertension agents. Finally, we compare our method to methods based on tabulation.

**Results:**

Our results show that regression methods provide more reasonable and useful results than tabulation, and successfully distinguish between medications that treat hypertension and medications that do not. These methods also provide insight into in which circumstances certain drugs are preferred over others.

**Conclusions:**

Our method can be used by health care institutions to monitor physician prescribing patterns and ensure the appropriateness of treatment.

## Background

An institution should monitor physician prescribing patterns to ensure that high-quality care is being provided at reasonable cost [1]. To accomplish this, the medications that patients are prescribed, and the conditions for which they are prescribed, would ideally be known. However, this information is often not available to an institution. Patients often visit multiple doctors at different institutions, and often their medications are not recorded in a centralized fashion. In addition, many medications have multiple indications, and the reasons that a medication is prescribed to a patient may not be recorded in a computable fashion in the patient’s electronic health record. Even if a medication and the reason for prescribing it are mentioned in the same note, typically mentions of conditions and medications are in separate sections of the notes and are not linked [2]. In some facilities, however, physicians do link medications to the reasons for prescribing them [3].

In the absence of a record that can be used to directly link medications and conditions, statistical methods can be used to try to determine, with respect to the population served by a health center, the medications that are used to treat conditions using data from the electronic health record (EHR). The simplest such statistical method would be the tabulation of the number of patients with a particular condition that are treated with a particular medication [1]. This method has been used in numerous studies of prescribing patterns, including of antibiotics, antihypertensives and antiasthmatics [4], but it normally requires a medical professional first to draw up a list of medications of interest. Some studies have also used regression to associate conditions and medications, although normally only taking into account general characteristics of the patients, like age and socioeconomic status, and also with a pre-selected list of medications [5]. Here, by contrast, we propose regression methods that can be used to determine, from among all medications used at the institution, those medications which are associated with a condition, while taking into account other conditions and medications recorded in the EHR in order to remove spurious associations and reveal the patient factors that influence the use of different medications.

The methods proposed here can be used to determine on a population level the medications used to treat a condition at an institution and the comorbidities that influence whether one class of medication might be used over another to treat a condition. These methods are related to methods we have previously presented and validated in the context of detecting adverse drug reactions (ADRs) using electronic health records [6]. Detecting ADRs using EHR data is a more difficult task than detecting treatments, for the association between a medication and the condition it treats is much stronger than the association between a medication and its side effects, which are generally rare. In addition, it is easier to validate a method for determining which medications are used to treat conditions, since adverse effects of medications may be unknown, whereas the medications used to treat conditions are generally known, for example, from drug labels and knowledge bases (like MEDI [7, 8]), although these sources are usually not complete [9]. The methods proposed in this paper constitute a comprehensive way to monitor institution-specific physician prescribing patterns, which can be used to improve the quality and cost-effectiveness of care being provided, and represent an improvement over simpler methods like chart reviews, tabulation and regression without control for comorbidities.

## Methods

In this study we restrict attention to outpatients cared for in the AIM primary care clinic on the Columbia University Medical Center (CUMC) campus of NewYork-Presbyterian/Columbia University Medical Center (NYP). Approximately 92% of the patients in the AIM clinic are on Medicaid or Medicare, and some of them lack insurance. We use EHR data for these patients from NYP, with approval from the CUMC Institutional Review Board. EHR data used consists of structured medication and conditions data obtained using the MedLEE natural language processing system [10] from outpatient notes. MedLEE identifies drug names (e.g., Lipitor) in narrative text in notes and uses RxNorm [11] to normalize them to their generic names (in the case of Lipitor, atorvastatin). MedLEE also identifies diseases and maps them to Unified Medical Language System concept identifiers to standardize them [12]. MedLEE identifies modifiers associated with diseases such as time and negation, so events not experienced by the patient, or experienced in the past, can be excluded.

We use medications and comorbidities extracted from the EHR for this analysis because the EHR provides a comprehensive view of a patient’s medical characteristics as opposed to, for example, ICD9 or ICD10 codes, which are often incomplete and of poor accuracy [13], or prescription orders, which will omit prescriptions ordered by doctors outside the institution. Outpatient notes at CUMC typically contain a list of medications taken at home, including over-the-counter medications.

**Fig. 1:**
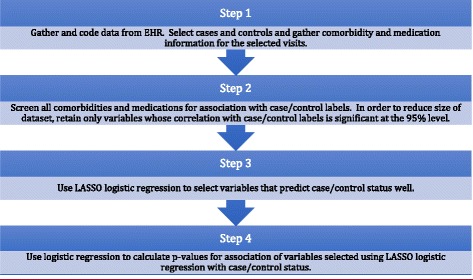
Outline of statistical procedure

We use a four-step statistical procedure, illustrated graphically in Fig. 1, to determine the medications that are used to treat a condition. Our first step is to create a case/control dataset. We use as cases outpatients that have ever had the condition of interest, and as controls we use outpatients that have never had the condition of interest. Then for cases we select one random outpatient visit when the condition of interest was recorded in the EHR. We also select one random outpatient visit for each control. For all the selected visits we record all the medications and conditions mentioned in the EHR, except for the condition of interest, which has already been captured in the case/control labels. This results in a dataset with only binary variables, each recording whether the patient has or does not have a condition or medication mentioned in the EHR for the applicable visit. Table 1 shows information about cases and controls for the case study of hypertension presented here, where we define cases as outpatients that have ever had hypertension and controls as outpatients that have never had hypertension. As can be seen in that table, patients with hypertension have more outpatient visits than patients without hypertension, and more unique conditions and medications recorded in the EHR.

**Table 1:** Summary statistics for hypertension case/control dataset

	Cases	Controls
Number of patients	46,722	76,391
Mean number of visits per patient (sd)	12.4 (13.5)	5.3 (6.5)
Number of unique conditions recorded per patient in all visits (sd)	30.3 (21.0)	13.4 (12.7)
Number of unique medications recorded per patient in all visits (sd)	17.7 (14.9)	10.4 (9.9)
Number of unique conditions recorded per patient for selected visit (sd)	13.1 (7.8)	4.9 (4.0)
Number of unique medications recorded per patient for select visit (sd)	5.9 (4.8)	3.8 (3.6)

In the second step of our statistical procedure, we screen all medications and conditions for association with the case/control labels. We calculate the Pearson correlation of each medication or condition variable with the case/control labels and retain all variables whose correlation with the case/control labels is significant at the 0.05 level. This is done to reduce the size of the dataset used in subsequent steps. We also remove variables that are perfectly correlated with one another. This can occur, for example, if two labels are always used by MedLEE to refer to the same condition or medication (for example, the input term *cystic kidney* maps to the UMLS code C1691228, corresponding to “cystic kidney disease” and also to the UMLS code C0022679, corresponding to “cystic kidney”; similarly *influenza vaccine* maps to C0770694, corresponding to “trivalent influenza vaccine” and also to C0021403, corresponding to “influenza virus vaccine”).

In the third step of our statistical procedure, we use LASSO logistic regression, a commonly used variable selection technique, to select among the variables retained in the second step those variables that predict case/control status well [14]; this step will remove variables that are not associated with case/control status, like medications that are used to treat other conditions and conditions that aren’t associated with the condition of interest.

In logistic regression we pose the following model. Let the response variable Y be 1 if a patient is a case, and 0 otherwise. Let the vector X encode all the conditions and medications of a patient. We model Pr(Y = 1|X = x) = exp.(ß_0_+ ß^T^x) / (1 + exp.(ß_0_+ ß^T^x)), where ß_0_ is an intercept coefficient and ß is a vector of other coefficients for each of the conditions and medications encoded in X. Here the interpretation of any element of ß is the logarithm of the odds ratio that the corresponding medication or condition is present in a case patient as opposed to a control patient.

Throughout this paper we use logistic regression models in which we include only main effects in the vector X, i.e., these models do not include any interactions among the condition and medication variables we extracted from the EHR. Interactions are variables encoding the presence of a combination of two or more variables together in the same individual (for example, diabetes mellitus and coronary heart disease). By omitting these interaction variables, we are implicitly assuming that the effect of a condition or medication on the log odds of case/control status for an individual is the same regardless of the presence or absence of any other condition or medication in that individual. We make this simplifying assumption because considering interactions would dramatically increase the number of possible variables to screen in our procedure.

LASSO logistic regression carries out logistic regression while requiring that the sum of the absolute values of the regression coefficients be less than the value of a given threshold. This constraint forces the values of smaller coefficients to zero. The lower the threshold, the more coefficients are forced to zero. The covariates with non-zero coefficients are the ones selected by the LASSO. We use 5-fold cross validation to select the value of the threshold. In 5-fold cross validation, the dataset is divided into 5 pieces. Each of these pieces in turn serves as a test dataset. Using only the other 4 pieces of the dataset, we fit the LASSO model with a sequence of threshold values and use the resulting fitted models (from different thresholds) to predict the case-control status of all the patients in the test dataset (which is not used in model fitting). The deviance, a statistical goodness of fit measure for logistic regression [15], is then calculated with respect to the test dataset for each threshold. The threshold we select is the highest threshold where the average deviance is within one standard error of the threshold at which the average deviance on the 5 test datasets is lowest. This is a common way to select the threshold, which results in selection of fewer covariates which are not good predictors [16]. We use the covariates with non-zero coefficients from the LASSO regression model with the selected threshold, fit using all of the dataset, in the final step of our statistical procedure.

Some medications that may be strongly associated with case/control status will nevertheless not be selected by LASSO regression, especially if very few patients take these medications. Table 2 shows the number of unique conditions and medications retained at each step of this procedure in the hypertension case study presented here. As can be seen in Table 2, the proportion of covariates recorded for small numbers of patients, both medications and conditions, decreases after each of our selection steps.

**Table 2:** Number of unique conditions and medications retained at each step of our statistical procedure for our hypertension case study

	# unique conditions in dataset	# unique conditions recorded for fewer than 20 patients	# unique medications in dataset	# unique medications recorded for fewer than 20 patients
After step 1 (creation of dataset with 1 visit per patient)	7601	5838	2321	1464
After step 2 (association screening)	1844	598	782	155
After step 3 (LASSO)	396	75	275	36

In the fourth step of our statistical procedure, we use logistic regression to calculate *p*-values for the association of those variables selected in the third step with case/control status. The results of this fourth step, after filtering, include the medications determined by our method to be likely used to treat the condition of interest.

To determine the comorbidities that influence whether one class of medication might be used over another to treat a condition, we first restrict our dataset to those patients with the condition of interest and that have been treated with one of the medications of interest. We then select for each patient a random outpatient visit when the condition of interest and one of the medications in the medication classes of interest was recorded in the EHR. A patient may be taking medications from more than one of these classes of medications. We therefore determine an outcome for each patient by randomly selecting among the classes of medications of interest recorded in the EHR for each patient for the selected visit.

With this dataset, we then screen for comorbidities significantly associated with the outcome and then carry out LASSO multinomial logistic regression to determine comorbidities that predict which class of drugs will be administered. Here we only include comorbidities, not medications, as explanatory variables, as our interest is in determining the comorbidities that affect which medication is used to treat a condition of interest. As before, we only include main effects in this regression model, i.e., we do not include any interaction variables.

In multinomial logistic regression we use the following model. If the response variable C (the class of drug prescribed) has K levels, and X encodes all the comorbidities of a patient, we model Pr(C = c|X = x) = exp.(ß_0c_ + ß_c_^T^x) / (exp(ß_01_+ ß_1_^T^x) + … + exp.(ß_0K_+ ß_K_^T^x)), where ß_0c_ is the intercept coefficient for the cth class and ß_c_ is the vector of other coefficients for the cth class. Suppose, as in our application, that two classes are A2 blockers and beta blockers and that the coefficients for the two classes for asthma are 0.018 and −0.043, respectively. To interpret these coefficients, we pick a reference category, beta blockers, for example. We then subtract the coefficient for beta blockers from the other coefficient, yielding 0.061. Then we exponentiate, yielding 1.06. This is the estimated factor by which asthma increases the odds of being prescribed an A2 blocker as opposed to a beta blocker. It is computationally expensive to calculate *p*-values for multinomial logistic regression with high-dimensional data like ours, so we do not do so. To rank comorbidities, instead of a *p*-value we use the LASSO threshold at which a variable is no longer selected.

As a comparison to the method described above, we also use tabulation to determine which medications are associated with hypertension. We first create a 2-by-2 table summarizing the occurrence of the 4 possible combinations of medication use/no medication use and outcome/no outcome. Then we calculate p-values for the null hypothesis that the odds ratio relating medication use to the outcome is 1 using Fisher’s exact test.

To evaluate our results, we compare medications detected using our method and the naïve tabulation method to medications listed in the MEDI ensemble medication indication resource, a compendium of medication-indication pairs from four publicly available resources: RxNorm, MedlinePlus, SIDER 2 and Wikipedia [7, 8]. We use the MEDI high precision subset, a set of high confidence medication-indication pairs present in either RxNorm or at least 2 of the 3 other indication resources. The MEDI indication resource and others like it specify indications for medications but do not, unlike our method, describe the actual use of medications in a specific population, including the preferential use of certain medications in patient populations with specific co-morbidities.

All statistical analyses were conducted in R.

## Results

Here we present use cases consisting of example analyses showing the utility of the methods described above, and focus on medication treatment of hypertension. First, we use regression to try to find the medications that are used to treat hypertension at the CUMC campus. Second, we determine the comorbidities that influence whether one class of hypertensive agents is used in preference over another class at the CUMC campus.

### Hypertension

We used our method to determine the medications that are used to treat hypertension at the CUMC campus. For comparison, we also used a naïve tabulation method. In both cases, we checked if the medications that were detected are listed in the MEDI indications-medication database as being indicated for hypertension. Results are shown in Table 3, for our method, and in Table 4, for the naïve tabulation method.

**Table 3:** Thirty drugs most highly associated with hypertension by logistic regression, sorted by *p*-value and, in the case of ties, odds ratio

Drug name	Adjusted OR	*p*-value	In MEDI?
Hydrochlorothiazide	22.3	0	T
Lisinopril	11.1	2.24e-287	T
Amlodipine	13.6	1.72e-243	T
Atenolol	8.03	3.03e-68	T
Metoprolol	3.10	1.51e-45	T
Enalapril	6.25	5.45e-45	T
Valsartan	6.71	7.71e-43	T
Chlorthalidone	33.4	1.12e-42	T
Labetalol	10.9	1.45e-40	T
Nifedipine	7.12	1.33e-35	T
Losartan	4.94	2.1e-35	T
Diltiazem	4.73	4.53e-18	T
Ramipril	5.45	2.58e-17	T
Sodium chloride	1.73	3.04e-14	F
Warfarin	1.83	4.4e-12	F
Telmisartan	4.42	3.02e-06	T
Progesterone	2.17	7.28e-06	F
Olmesartan	3.66	7.72e-06	T
Aspirin	1.22	4.24e-05	F
Verapamil	2.52	4.82e-05	T
Benazepril	3.53	9.31e-05	T
Norethindrone	1.40	0.000211	F
Carvedilol	1.56	0.000324	T
Probenecid	2.05	0.000444	F
Candesartan	6.87	0.000733	T
Bisoprolol	33.4	0.00123	T
Torsemide	6.54	0.00157	T
Megestrol	3.62	0.00293	F
Methyldopa	10.8	0.00312	T
Nebivolol	6.15	0.00329	T

The adjusted OR is the estimated adjusted odds ratio for use of the drug comparing those with and without hypertension (adjusted for all other variables included in the regression model)

**Table 4:** Thirty Drugs most highly associated with hypertension by naïve tabulation, sorted by *p*-value and, in the case of ties, by odds ratio

Drug name	Unadjusted OR	*p*-value	In MEDI?
Hydrochlorothiazide	43.1	0	T
Amlodipine	31.4	0	T
Lisinopril	29.8	0	T
Losartan	23.9	0	T
Atenolol	21.1	0	T
Valsartan	19.9	0	T
Metoprolol	14.8	0	T
Hepatitis b antigen peptide	13.0	0	F
Glipizide	12.6	0	F
Carvedilol	12.4	0	T
Simvastatin	9.90	0	F
Atorvastatin	9.76	0	T
Aspirin	9.64	0	F
Metformin	8.30	0	F
Furosemide	7.69	0	T
Insulin	7.09	0	F
Omeprazole	3.62	0	F
Multi vitamin	2.91	0	F
Chlorthalidone	69.7	2.83e-319	T
Clopidogrel	10.6	4.29e-317	F
Calcium	3.20	5.62e-298	F
Glucose	3.31	8.85e-271	F
Esomeprazole	3.23	1.88e-262	F
Rosuvastatin	7.94	2.12e-254	F
Nifedipine	17.0	9.32e-251	T
Ergocalciferol	3.23	1.32e-246	F
Alendronate	6.57	1.46e-246	F
Warfarin	4.25	1.9e-222	F
Enalapril	10.8	2.79e-222	T
Docusate	2.88	9.2e-214	F

The unadjusted OR is the estimated odds ratio for use of the drug comparing those with and without hypertension. The *p*-value comes from Fisher’s exact test

One way to filter the results is to sort by *p*-value and, in the case of ties, the odds ratio. Ties occur for the tabulation method since many of the *p*-values from Fisher’s exact test are indistinguishable from zero. As shown in Tables 3 and 4, results using regression are substantially more likely to be in the MEDI high precision hypertension subset. They are therefore substantially more likely to actually have been prescribed for hypertension. Out of the top 30 medications for our method, 23 are in MEDI, whereas only 13 are for the naïve tabulation method. We have included the top 30 medications here, but the performance of our method is at least as good as MEDI for all thresholds less than 30 as well.

In lieu of a fixed threshold like 30, a threshold could be selected using the false discovery rate, for example, which is designed to control the proportion of discoveries that are false among all discoveries [17]. For example, if we fix a false discovery rate of 5%, then using the Benjamini-Hochberg procedure for adjusting *p* values to control the false discovery rate [17], our method discovers 53 drugs used to treat hypertension, of which 30 (56.6%) are in MEDI. By contrast, the naïve tabulation method discovers 169 drugs used to treat hypertension, of which 38 (22.5%) are in MEDI. In both cases, if p values were properly calibrated and if MEDI included all drugs used to treat hypertension, we would expect to see 95% of the drugs with an adjusted *p* value less than 5% in MEDI.

### Comparison of hypertension drugs

With expert physician assistance, we divided the most commonly used hypertension drugs at the CUMC campus into five different classes. The drugs and classes, and the numbers of hypertension patients with a drug from each class mentioned in their note, are listed in Table 5. These numbers do not correspond exactly to the numbers of hypertension patients with prescriptions for these drug classes since drugs are often mentioned in notes even if they are not being currently prescribed, and MedLEE cannot always distinguish between currently prescribed drugs and other drugs. The most common class of drugs mentioned in the notes is thiazides, followed by ACE inhibitors. It is very common for more than one class of drugs to be mentioned in a note; only 41.5% of the notes for patients with hypertension that mentioned one of these classes of drugs mentioned exactly one of the classes. Therefore, many of the 21,518 patients that we analyze are represented in more than one cell of this table.

**Table 5:** Hypertension medications included in each class, as well as the number of patients with a member of that class mentioned in their note

Drug class	Drugs	Number of patients
A2Blocker	Valsartan, losartan, telmisartan, olmesartan	3993
ACE inhibitor	Lisinopril, ramipril, enalapril, benazepril	9877
Beta blocker	Carvedilol, labetalol, atenolol, metoprolol	8327
Calcium channel blocker	Amlodipine, nifedipine, diltiazem	7539
Thiazide	Chlorothiazide, hydrochlorothiazide, chlorthalidone	10,239

We used our method to find comorbidities that influenced which of these drug classes would be prescribed for hypertension. The top thirteen such conditions are shown in Table 6. From this table we see, for example, that patients with asthma are less likely to be prescribed a beta blocker for their hypertension, and that patients with stage 5 chronic kidney disease are less likely to be prescribed a thiazide.

**Table 6:** Comorbidities associated with which class of hypertension medication is prescribed

	A2Blocker	ACE	Beta_Blocker	Cal_Chan	Thiazide	Percent of patients
Asthma	0.018	−0.014	−0.043	0.009	0.030	15.2
Bestrophinopathy	0.391	−0.248	−0.081	−0.120	0.058	1.7
Cardiomyopathies	−0.013	0.029	0.079	−0.041	−0.054	3.1
Chronic kidney disease stage 5	−0.011	−0.030	0.068	0.061	−0.089	1.7
Congestive heart failure	0.012	0.073	0.416	−0.071	−0.429	5.7
Coronary heart disease	−0.023	−0.057	0.481	−0.091	−0.310	11.2
Diabetes mellitus	0.101	0.261	−0.042	−0.122	−0.197	45.7
Heart failure	0.003	−0.017	0.069	0.005	−0.060	2
Hypertension induced by pregnancy	−0.017	−0.037	0.044	0.047	−0.038	0.5
Ischemia	−0.006	−0.004	0.077	−0.013	−0.055	3.8
Kidney failure	−0.022	−0.121	0.161	0.200	−0.217	9.6
Renal insufficiency	−0.008	−0.038	0.056	0.058	−0.067	8.6
Tricuspid valve insufficiency	0.000	−0.001	0.004	0.001	−0.004	3.7

To interpret any coefficient, first pick a reference class from among the five classes of medications. Then subtract the coefficient for any comorbidity for the class of interest from the coefficient for that comorbidity for the reference category and exponentiate. This is the estimated factor by which the comorbidity increases the odds of being prescribed the class of interest as opposed to the reference class. Positive coefficients are colored green and negative coefficients red, and shaded darker if the absolute value of the coefficient is greater than 0.05

## Discussion

We have presented methods for monitoring physician prescribing patterns within an institution. As measured by a comparison with results from MEDI, performance is superior to a naïve tabulation approach that does not take into account other conditions or medications of patients.

Our approach is able to produce a snapshot of an institutional approach to treating hypertension. Patients at the CUMC campus are on one of the five classes of antihypertensive agents recommended in the most recent guidelines from the Eighth Joint National Committee [18]. Furthermore, nearly half of patients are on a thiazide diuretic, a recommended first-line treatment for hypertension given its efficacy and low cost. A similar study in China, using a less automated approach, demonstrated that thiazides were being underutilized [1].

The snapshot also reveals appropriate usage in particular comorbid conditions. For example, treatment of hypertension during pregnancy should avoid the potentially teratogenic ACE inhibitors and A2 receptor blockers. Rather, beta-blockers are preferred as they are thought to be safe. Table 6 demonstrates that this guideline is followed at the CUMC campus.

Some of the medications that our method determined are used to treat hypertension, like warfarin and progesterone, are actually being used to treat other conditions, and are likely indirectly associated with hypertension since those other conditions are more common among patients with hypertension. This method is therefore subject to confounding, or spurious associations between medications and conditions that arise because some relevant variables are not fully taken into account, perhaps because they are not adequately reflected in the EHR. The failure to completely control for confounding results in *p*-values that are too low, and in too many false discoveries, as illustrated by our comparison of the *p*-values generated from our method, as adjusted for false discoveries, with the MEDI database.

Extensions of this method could be used to study, for example, for which conditions a certain medication is prescribed. For example, a medical center could determine which conditions lead to an opiate prescription. The results of such an analysis could be used to suggest conditions for which opiate overprescription is a problem.

There are several limitations to this method. The clinical notes which we use are incomplete and do not include all relevant information, like, for example, information on when exactly patients started and stopped taking each relevant medication and developed each relevant comorbidity. Moreover, clinical notes can be difficult to interpret, and may be misinterpreted by the MedLEE NLP system. Further work should apply these methods to additional conditions, to different patient populations, and also to data from other facilities, as there may be differences in data quality between facilities that should be taken into account. Further work could also explore the value of matching patients, or visits of patients, between cases and controls, which could further reduce the effect of confounding.

## Conclusions

We have presented a useful set of methods to analyze physician prescribing patterns at an institution. They enable an institution to analyze for which conditions medications are being prescribed, how conditions are being treated, the appropriateness of treatments, and in which circumstances certain drugs are preferred over others.

## Abbreviations

ADR: Adverse drug reaction
CUMC: Columbia University Medical Center
EHR: Electronic health record
NYP: NewYork-Presbyterian/Columbia University Medical Center
